# Gradient Distribution of Martensite Phase in Melt-Spun Ribbons of a Fe-Ni-Ti-Al Alloy

**DOI:** 10.1186/s11671-016-1313-0

**Published:** 2016-02-20

**Authors:** Volodymyr Bondar, Vitalij Danilchenko, Ievgenij Dzevin

**Affiliations:** G.V.Kurdyumov Institute of Metal Physics, NAS of Ukraine, Vernadsky Blvd. 36, Kyiv, 03680 Ukraine

**Keywords:** Iron–nickel alloys, Rapid melt quenching, Melt-spun ribbon, Martensitic transformation, Grain size effect, Phase hardening

## Abstract

Metallographic, X-ray diffraction and magnetometric analysis were used to study the regularities of martensitic transformation in melt-spun ribbons of a Fe – 28 wt. % Ni – 2.1 wt. % Ti – 2 wt. % Al – 0.05 wt. % C alloy. The substantial differences in volume fractions of the martensite phase in local regions of thin melt-spun ribbons of the alloy are related to the size effect of the transformation and structural inhomogeneity of the ribbons. The distribution of austenitic grain size in different local areas of melt-spun ribbons is significantly different. The principal factor for changing the completeness of the martensitic transformation is the size effect of transformation. Difference in the martensite volume fraction in local regions of a ribbon is mainly determined by the different volume fractions of ultrafine-grained (500–1000 nm) and nanosized (80–100 nm and less) initial austenite grains, in which the transformation was slowed down or completely suppressed. Other factors almost do not affect the completeness of the martensitic transformation. The strong stabilizing effect of the reverse α–γ transformation with respect to the subsequent direct γ–α transformation in the melt-spun ribbons is also related to the grain size effect.

## Background

The new materials with a variety of significantly improved physical and mechanical properties, which are determined by phase composition and microstructure that are formed in the non-equilibrium conditions of solidification and crystallization, can be obtained by quenching from the melt. Additional possibilities for controlling structure and properties of rapidly quenched alloys are offered by phase transformation, as phase transformations are sensitive to the cooling and heating rates of alloys, grain size of the initial phases, and residual stresses. The formation of the martensitic α-phase is influenced by a grain size effect, the redistribution of alloying elements, and residual stresses in the initial austenite. Martensitic transformations have been investigated, for example, in powders of Fe-based alloys, in nanocrystalline particles of γ-iron in a copper matrix, and in ultrafine grains generated by intensive plastic deformation [1–4] and quenching from the melt [5–7].

The formation of desired physical and chemical properties of ultrafine-grained metastable alloys prepared by melt quenching is to a large extent related to the possibility of the controlling characteristics of martensitic transformations which can occur during alloy solidification and subsequent cooling and under the action of external factors. Prospects for the development of new materials based on rapidly quenched Fe–Ni alloys, in which the direct γ–α and reverse α–γ martensitic transformations are realized during cooling and subsequent heating, are considered. The controlled martensitic transformations in such thin ribbons can be used for strengthening, realization of shape memory effects, improvement of magnetic (hysteretic) properties, regulation of thermal expansion coefficient, etc. The martensitic transformations can be used in measure for the formation of a new combination of physical, mechanical, and functional properties of rapidly quenched metastable alloys only as a result of systematic studies of transformation features that are due to specific non-equilibrium conditions of rapid melt quenching. In the case of occurrence of the phase transformations in thin ribbons of metastable alloys, the inhomogeneity of their structural state can cause substantial changes in characteristics of the transformations. This can lead to changes in the physical and mechanical properties in local regions of the material.

For this paper, we have investigated the γ–α martensitic transformation in the local regions of the ribbon in the Fe – 28 wt. % Ni – 2.1 wt. % Ti – 2 wt. % Al – 0.05 wt. % C alloy which was quenched from the melt. Those features were caused by a significant difference in the rate of cooling of the near-surface layers on the contact surface (i.e., the surface of the ribbon in contact with the cooling disk) and the free surface (i.e., the surface of the ribbon opposite to the cooling disk).

## Methods

The alloy used in the present work had the composition Fe – 28 wt. % Ni – 2.1 wt. % Ti – 2 wt. % Al – 0.05 wt. % C. A melt-spun ribbon of the alloy with a thickness of 40–50 μm and a width of about 8 mm was produced by the melt-spinning method in a carbon dioxide atmosphere. The continuous ribbon was produced by the melt-spinning technique from 100 g of the alloy. The ribbon was in the austenitic state at room temperature. Martensitic γ–α transformation in the ribbon was realized by cooling in liquid nitrogen. The temperature intervals of martensitic transformations in bulk alloy are direct γ–α transformation 253–123 K and reverse α–γ transformation 893–1013 K. The temperature intervals of martensitic transformations in the melt-spun ribbon are direct γ–α transformation 218–113 K and reverse α–γ transformation 863–933 K.

The phase composition of the ribbon was determined using a DRON-3 X-ray diffractometer with FeK_α_ radiation by means of the method was proposed in Ref. [8]. For the investigated alloy, the austenitic planes (111) and martensitic planes (110) are parallel to each other. The amount of martensitic phase was calculated using the following equation:

$$ M = \left({I}_{\left(\mathrm{h}\mathrm{k}\mathrm{l}\right)\mathrm{M}}/\ {P}_M\right)\ \mathrm{x}\ \left(100\ /\ \left(\left({I}_{\left(\mathrm{h}\mathrm{k}\mathrm{l}\right)\mathrm{M}}/\ {P}_M\right) + \left({I}_{\left(\mathrm{h}\mathrm{k}\mathrm{l}\right)\mathrm{A}}/\ {P}_A\right)\right)\right), $$

where *I*_(hkl)A_ and *I*_(__hkl)M_ are the integral intensity of the diffraction lines from the lattice planes of austenite and martensite, respectively. *P*_*A*_ and *P*_*M*_ are the multiplicity factors of the lattice planes concerned. In the present case, *P*_*A*_ = 8 and *P*_*M*_ = 12 for the planes (111)_γ_ and (110)_α_. This equation can be used for all planes of austenite and martensite provided that the material is free of preferred orientation. However, in the present case, the investigated ribbon exhibited considerable texture. For example, the ratio of the intensities of the austenitic diffraction lines *I*_200_/*I*_111_ was 4.5, whereas a value of 0.5 is expected for an untextured polycrystalline material. Therefore, only reflections of austenite and martensite pertaining to planes that are parallel to each other in accordance with the orientation relationship can be used for calculating the amount of martensitic phase.

## Results and Discussion

The investigated alloy was microcrystalline and has an austenitic structure at room temperature.

The grain sizes are different owing to the different solidification rates of the surface layers and the ribbon volume. In local areas of the ribbon, structure is significantly heterogeneous and different. Due to the different crystallization rates of near-surface layers and volume, the size of grains was different for those areas. Smaller grains were formed at the contact surface than that on the free surface. In the volume of the ribbon, grains had intermediate size. The grain size of the ribbon also varied over the width; in the central part it was smaller than on the peripheral part of the ribbon (edge effect). Results of investigation of initial austenite microstructure on specimens that were cut from different points of the ribbon led to the conclusion that the grain size increase from head to the end. The size of grain at the contact surface was 0.1–3 μm and at the free surface 0.1 – 4 μm. Some part of grains was nanosized—from 0.1 to 0.05 μm.

**Fig. 1:**
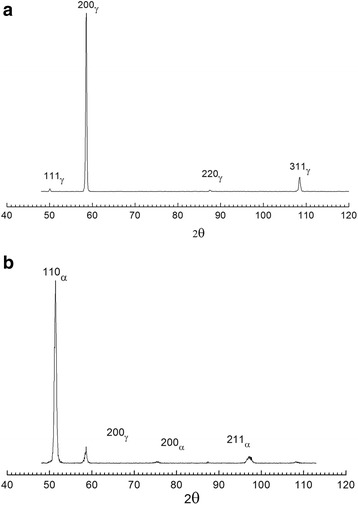
X-ray diffraction *patterns of free* surface of the ribbon. **a** Initial state. **b** After cooling in liquid nitrogen

X-ray diffraction measurements have shown that the austenitic phase on the free surface formed with a pronounced (100)γ growth texture (Fig. 1а). This texture was significantly less developed on the contact surface. The degree of texture deduced from the integral intensities of the reflections (111)γ and (200)γ changed along the width and length of the ribbon in accordance with the change of the cooling rate during the crystallization.

**Fig. 2:**
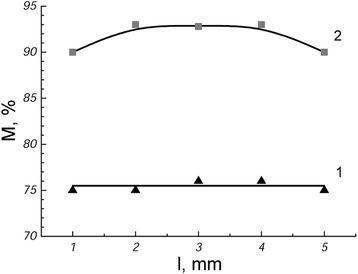
The amount of martensite at the contact (*1*) and free (*2*) surfaces of the ribbon

Intensive martensitic transformation occurs in the ribbon during cooling in liquid nitrogen (Fig. 1b). The amount of martensite was different for different sides of the ribbon (Fig. 2) and was at the free surface 83 %, whereas the amount at the contact surface was 55 %. In the central part of the free surface of ribbon, the amount of martensite was higher in comparison to the edges of the ribbon (edge effect). At the contact surface of the ribbon, only a weak edge effect was observed.

In [9], it was found that the volume fraction of martensite formed at the contact and free surfaces of the rapidly quenched Fe – 31.4 wt % Ni – 0.02 wt % C alloy ribbon is 95 and 60 %, respectively. The authors explained the different completeness of the transformation by enrichment of the 10-μm layer with nickel at the free surface.

Analysis of the variations of martensitic amount at different areas of our ribbon, through its width and length, showed that the difference in the completeness of the γ–α martensitic transformation is connected to the different average sizes of the austenite grains in these areas (grain size effect of transformation). The grain size effect implies the decrease of the start temperature of martensitic transformation and in the reduction of the amount of martensitic phase with the decreasing size of the initial austenitic grain. Earlier, the size effect of transformation was observed in bulk iron–nickel [10–11] and in thin ribbons of rapidly quenched alloys [12]. Below a certain critical grain size, the martensitic transformation was completely suppressed in the austenite grain. This critical size declined from 3.5 μm in the Fe – 32 wt. % Ni alloy to 0.3 μm in the Fe – 29 wt. % Ni alloy [5].

In the grains of the rapidly quenched Fe – 28 wt. % Ni – 2.1 wt. % Ti – 2 wt. % Al – 0.05 wt. % C alloy with grain sizes between 1.3- and 4-μm, single crystals of martensite with needle morphology formed upon cooling. In most cases, the martensitic transformation during cooling in liquid nitrogen did not take place in grains smaller than 1.3 μm. Martensitic crystals are not formed in all investigated grains of the size 1.3 μm and smaller. In our experiments, the critical size of austenite grains of the Fe – 28 wt. % Ni – 2.1 wt. % Ti – 2 wt. % Al – 0.05 wt. % C ribbon was 0.8–1.3 μm. The amount of martensite decreases with decreasing size of austenite grains.

It is known that the intensity of martensitic transformation in iron–nickel alloys depends on their chemical composition and character of residual stresses. In this connection, we studied the distribution of alloyed elements in the ribbons. It was shown that the Ni content at the free surface of a ribbon is 0.5–1.0 % higher than that at the contact surface. Across the ribbon, the difference is lower. Fluctuations of the concentration of Ti and Al were even more insignificant. These insignificant variations of concentration cannot explain the observed difference in the amount of martensite in local regions of the ribbon.

Farther, we determined the residual stresses in local regions of the ribbon. The stresses were measured for the contact and free surfaces of ribbons across the whole width of the ribbon. The results of the measurements were averaged for different regions of the ribbon in the near-surface layers of which the formation of the diffracted beam occurred. To increase the locality of measurements of stresses, we used a URS002 X-ray unit equipped with a sharp-focus tube producing a focal spot of 30 μm in diameter. This allowed us to measure stresses in local regions, which could solidify at different cooling rates (central and peripheral zones, regions with different grain size distributions). It was found that at the contact surface, low tensile stresses close to the accuracy of measurements are formed during solidification. At the free surface, no residual stresses were present across the almost entire ribbon. The insignificant level of stresses is related to the fact that the temperature of the ribbon which came off from the disk surface was sufficiently high (773–873 K). At this temperature, the residual stresses, which can be induced during rapid solidification of the ribbon, relax in the course of preparation. We failed to ensure a more intense heat removal during melt spinning and, therefore, a higher quenching effect when using this technology. These measurements showed that residual stresses cannot markedly affect the distribution of the martensite volume fraction in thin melt-quenched ribbons.

Thus, the principal factor for changing the completeness of the martensitic transformation is the size effect of transformation. The difference in the amount of martensite in local regions of a ribbon is mainly determined by the different volume fractions of ultrafine-grained (0.5–1.0 μm) and nanosized (0.1–0.2 μm) initial austenite grains, in which the transformation was slowed down or completely suppressed. Indeed, an analysis of the microstructure of thin ribbons showed that the sizes of initial austenite grains correspond to the range of realization of the size effect of transformation. Other factors almost do not affect the completeness of the martensitic transformation.

## Conclusions

In different regions of the melt-quenched Fe – 28 wt. % Ni – 2.1 wt. % Ti – 2 wt. % Al – 0.05 wt. % C ribbons, differences in the size of austenite grains formed at the contact and free surfaces and across the ribbon are observed owing to different cooling rates. The amount of martensite formed during cooling depends on the austenite grain size. The completeness of the direct γ–α transformation is determined mainly by the volume fraction of ultrafine-grained and nanosized grains of the initial austenite (1.3–0.8 μm in size and less), in which the transformation is suppressed completely.
